# Spectrum of acute kidney injury associated with cocaine use: report of three cases

**DOI:** 10.1186/s12882-019-1279-0

**Published:** 2019-03-20

**Authors:** José Célio Costa Lima Filho, Maurício Yukio Ogawa, Tacilla Hanny de Souza Andrade, Sami de Andrade Cordeiro Gadelha, Paula Frassinetti Castelo Branco Camurça Fernandes, Anaiara Lucena Queiroz, Elizabeth De Francesco Daher

**Affiliations:** 1 Nephrology Training Program, Department of Nephrology, Walter Cantídio University Hospital, Fortaleza, Brazil; 2 Medical Sciences Graduate Program, Department of Internal Medicine, School of Medicine, Federal University of Ceará, Fortaleza, Brazil; 3 Medical Sciences Postgraduate Program, Department of Internal Medicine, School of Medicine, Estadual University of Ceará, Fortaleza, Brazil; 4 Pathology Department of Nephrology, Department of Pathology, Walter Cantídio University Hospital, Fortaleza, Brazil; 5 Department of Internal Medicine, School of Medicine, Federal University of Ceará, Fortaleza, Brazil

**Keywords:** Acute kidney injury, Cocaine use, Rapidly progressive glomerulonephritis, Thrombotic microangiopathy, Cocaine-induced rhabdomyolysis

## Abstract

**Background:**

The consequences of cocaine use are multisystemic, such as, for instance, renal failure, hepatotoxicity and pulmonary toxicity, with renal alterations being the focus of the present study. The use of substances that modify the base composition of cocaine (or adulterants) aiming to potentiate its effects also has an impact on these manifestations. The present study aims to report three cases with different diagnosis of acute kidney injury related to cocaine use.

**Case presentation:**

**Case 01 -** A 30-year-old female patient, who regularly used cocaine, started to have lower-limb edema, which showed a progressive and ascending evolution, affecting the face a few days later, associated with an isolated febrile episode and oligoanuria. The presence of cytoplasmic antineutrophil cytoplasmic antibodies (C-ANCA) was verified: reactive 1:80, with renal biopsy compatible with rapidly progressive glomerulonephritis (RPGN). **Case 02** - A 34-year-old female patient, with difficult-to-control hypertension and a frequent user of cocaine, showed generalized sudden edema together with diffuse and progressive pruritus associated with oliguria, fever, nausea, and vomiting. Schistocyte screening was positive, with negative direct Coombs test, and negative serologies for hepatitis B, C and HIV, as well as negative anti-double-stranded DNA, Anti-SSA and Anti-SSB. The renal biopsy was compatible with thrombotic microangiopathy, associated with moderate interstitial fibrosis and acute tubular necrosis **Case 03** - A 25-year-old male patient who had been a cocaine user for 5 years had a sudden onset of generalized disabling myalgia (especially in the lower limbs) associated with recent frontotemporal headache, palpitation, dizziness, and a non-measured febrile episode; the patient had used cocaine at the night before symptom onset. CPK was 1731 U/L.The final probable diagnosis was AKI secondary to cocaine-induced rhabdomyolysis**.**

**Conclusions:**

In conclusion basically, 05 etiologies of acute kidney injury should always be remembered: rhabdomyolysis, thrombotic microangiopathy, vasculitis, acute interstitial nephritis and renal infarction. Emphasis should be given to rhabdomyolysis due to its higher prevalence. Considering the increasing rates of cocaine use, especially with the use of adulterating substances, these pathologies will likely be increasingly prevalent.

## Background

The deliberate and harmful use of cocaine is currently prevalent worldwide and constitutes a significant public health problem [1]. Despite a drop in cocaine users between 2007 and 2014, the prevalence of users is significant with about 18.3 million users worldwide in 2014 [2]. In Brazil, the prevalence of consumption is hundreds of thousands, being one of the main countries in the total use of the substance [2]. There is a significant increase in cocaine consumption, despite a reduction in consumption in the United States and relative stability in Europe. The increasing commercialization of this substance in areas of socioeconomic fragility that has little access to a rehabilitation system is one of the justifications for this increase [3, 4].

The consequences of cocaine use are multisystemic, such as, for instance, renal failure, hepatotoxicity and pulmonary toxicity, with renal alterations being the focus of the present study. The use of substances that modify the base composition of cocaine (or adulterants) aiming to potentiate its effects also has an impact on these manifestations [5].

The present study aims to report three cases with different diagnosis of acute kidney injury related to cocaine use.

## Case presentation

### Case 01

A 30-year-old female patient, without comorbidities, who regularly used cocaine and marijuana, started to have lower-limb edema, which showed a progressive and ascending evolution, affecting the face a few days later, associated with an isolated febrile episode and oligoanuria. Initial laboratory examinations showed Hb of 9.44 g/dL; Ht of 26.8%; MCV of 93 fL; MCH of 30.7 pg; leukocytes 10,700mm^3^ (segmented 7597, eosinophils 428, lymphocytes 1926, monocytes 749); platelets 284,000mm^3^; Cr 14 mg / dL; Ur 225 mg / dL; Na + 135 mEq /L; K + 6 .5mEq / L; Serology for HIV, hepatitis B and C negative (Table 1).

**Table 1:** Result of major laboratory tests

	Case 1	Case 2	Case 3
Hb (g/dl) and Ht (%)	9,44 and 26,8	6,6 and 19,6	–
Urea (mg/dl)	225	227	259
Creatinine (mg/dl)	14	13,3	13,8
Creatine kinase (u/l)	–	128	1731 (Five days after admission)
Serology for HIV, Hepatitis B and C	Negative	Negative	Negative

Urinalysis showed pH: 5.0; UD: 1015, Proteins: +++ / 4; Leukocytes: +/− 4; Hemoglobin: +++ / 4. Sedimentoscopy: numerous red blood cells, numerous leukocytes, rare epithelial cells; presence of coarse granular cylinders; moderate bacteriuria; 24-h Proteinuria: 2161 mg (Volume: 400 mL / 24 h).

**Fig. 1:**
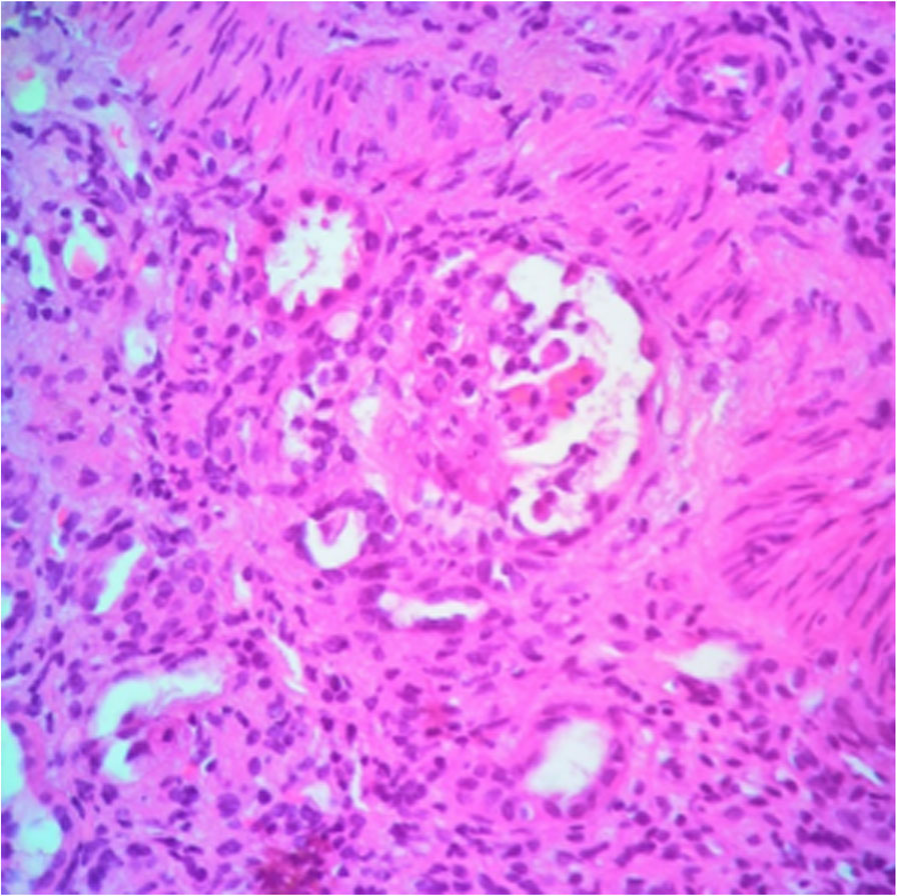
Glomerulus displaying cell crescent with ruptured Bowman’s capsule. (Hematoxylin & Eosin, 400×)

**Fig. 2:**
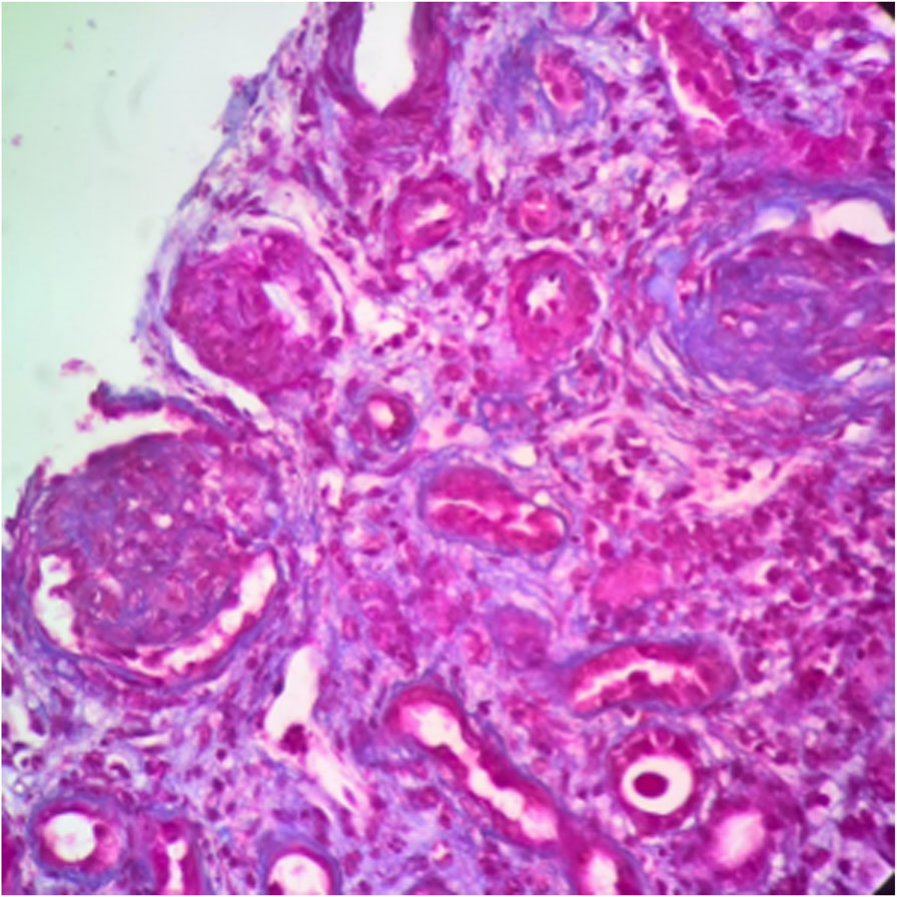
Glomerulus to the right displaying overall sclerosis. Glomerulus to the left displaying cell crescent. (Masson’s trichrome, 400×)

**Fig. 3:**
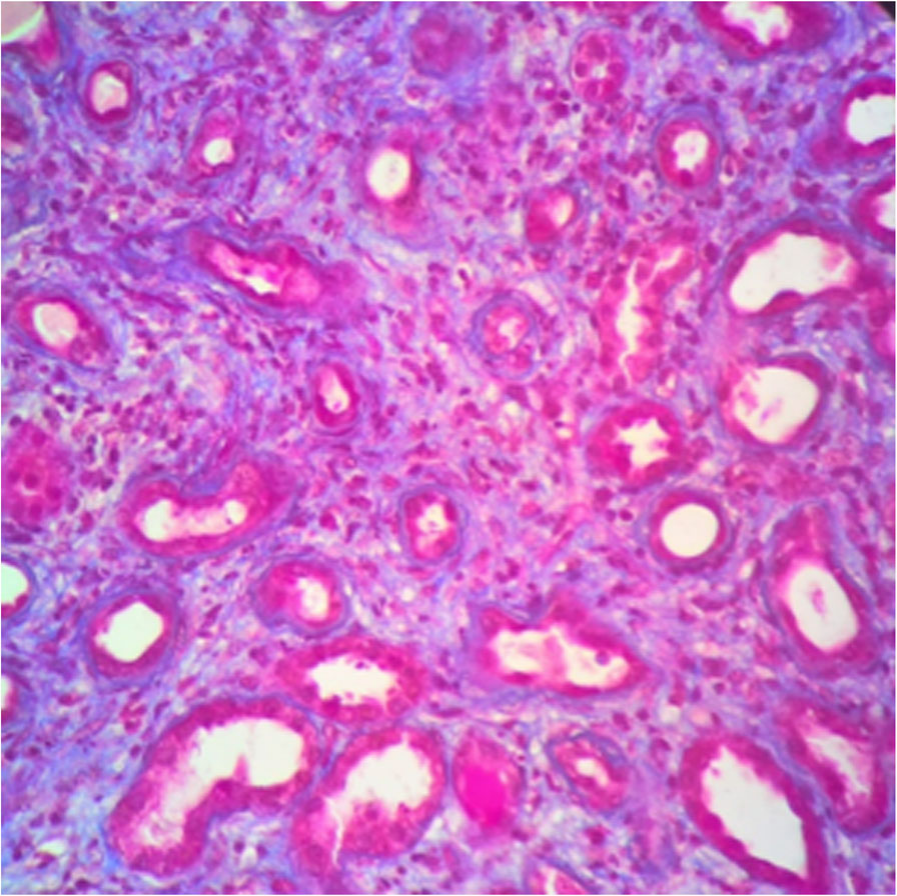
Tubular atrophy area. (Masson’s trichrome, 400×)

The presence of proteinase-3 31 IU/ml (Reference Value: reagent if > 3 IU/ml), cytoplasmic antineutrophil cytoplasmic antibodies (C-ANCA) was verified: reactive 1:80, with renal biopsy (Figs. 1, 2 and 3) compatible with rapidly progressive glomerulonephritis (RPGN), while immunofluorescence showed granular deposits in the capillary loops of C3c (one cross) and fibrinogen (one cross). The patient was hospitalized and submitted to pulse therapy with methylprednisolone 1 g / day for 3 days and cyclophosphamide IV. Her evolution showed no recovery of renal function, she abandoned treatment and remained in dialysis treatment. The diagnosis was pauci-immune ANCA-related RPGN.

### Case 02

**Fig. 4:**
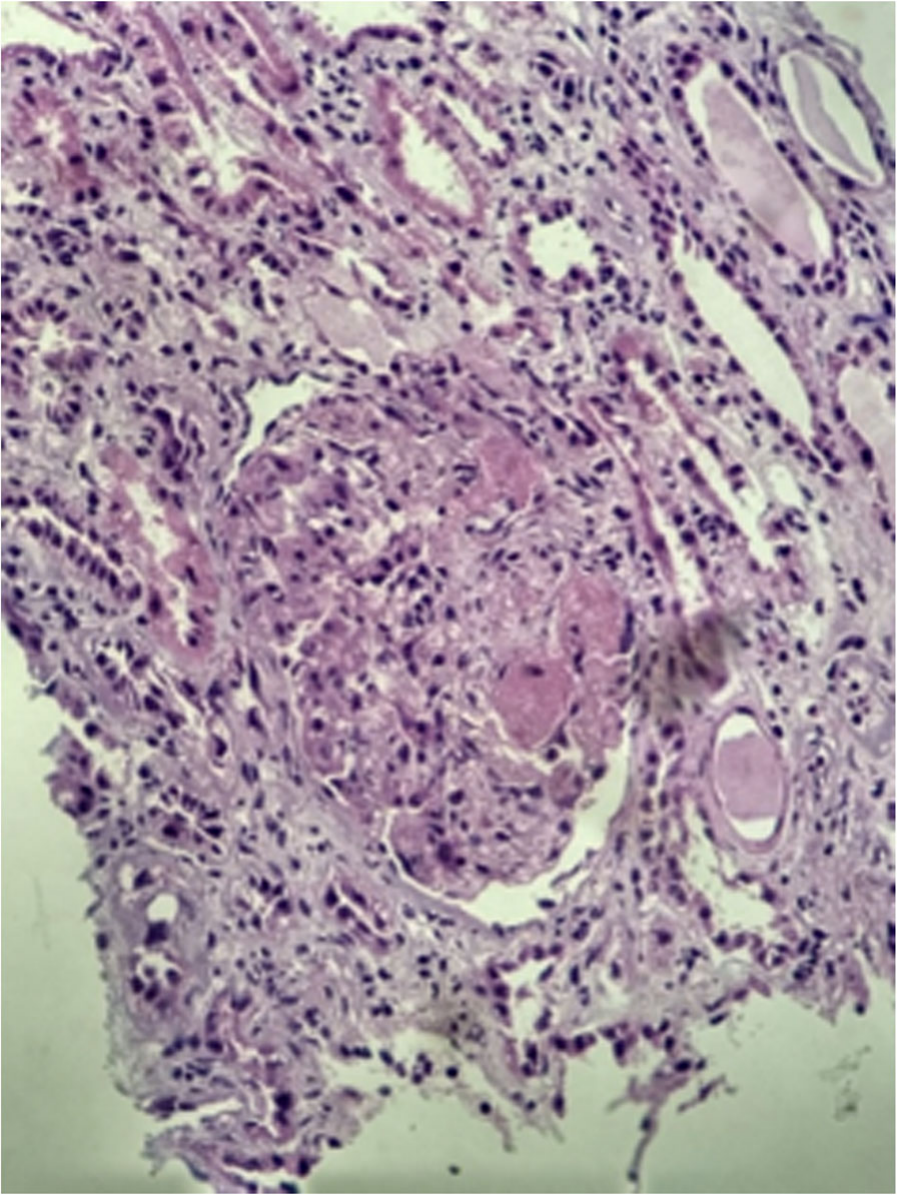
Glomerulus displaying microthrombi in glomerular capillary lumens. (Hematoxylin & Eosin, 400×)

**Fig. 5:**
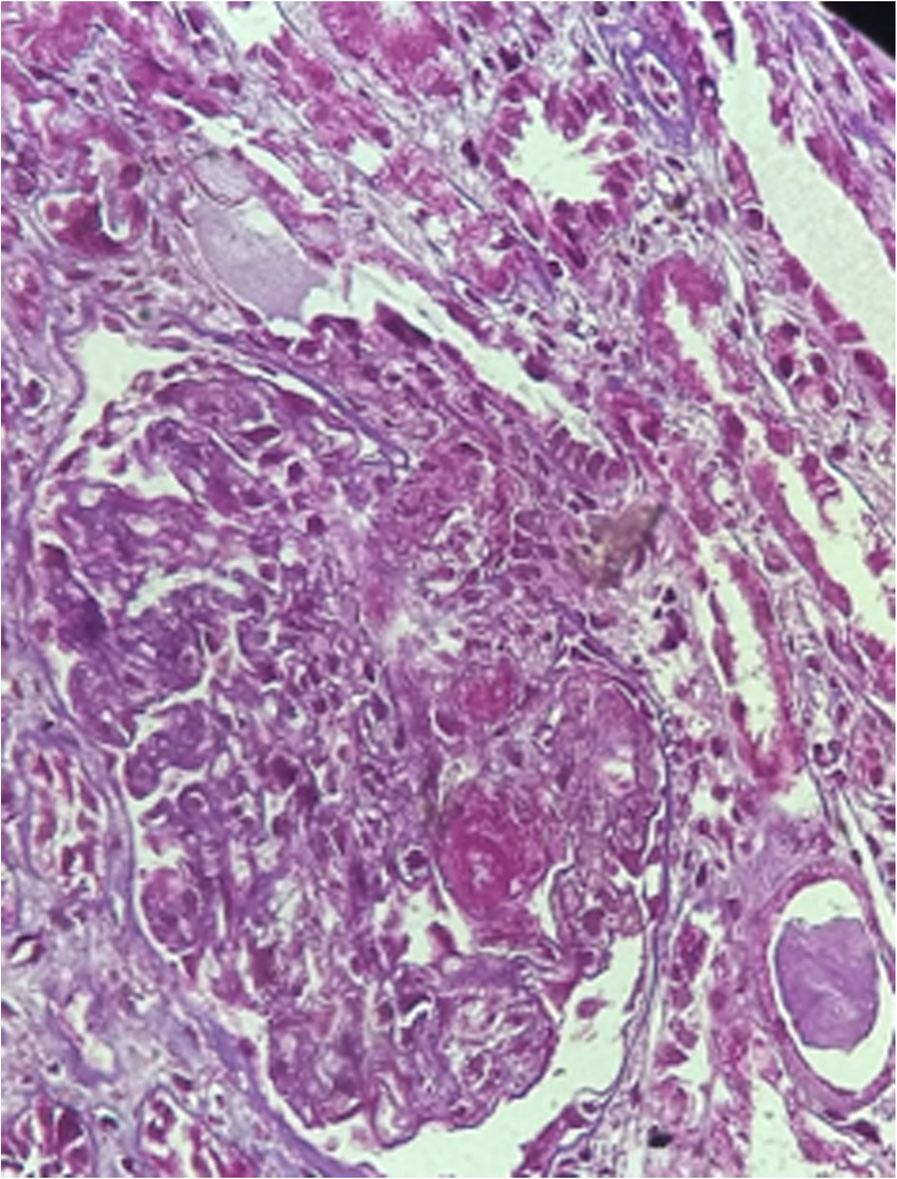
Microthrombi enhanced by Periodic Acid Schiff (PAS) staining (PAS, 400×)

A 34-year-old female patient, with difficult-to-control hypertension and a frequent user of cocaine, showed generalized sudden edema together with diffuse and progressive pruritus associated with oliguria, fever, nausea, and vomiting. The initial laboratory tests showed Hb of 6.6 g / dL; Ht of 19.6%; MCV: 91.1%; MCH of 30.6pg; RDW: 13.6%; Leukocytes: 9914mm^3^; platelets: 79150mm^3^; Reticulocytes 3.39%; LDH 2702 IU/L; TB 0.65 mg/dL; DB 0.21 mg /dL; IB: 0.44 mg /dL; Creatinine 13.3mg / dL; Urea 227 mg / dL; K+ 4.7 mEq /L; APTT 0.89 s; INR 0.87; CPK 128 u /L; C3 114 mg / dL; C4 26 mg / dL; Serology for HIV, hepatitis B and C negative. Urinalysis: pH: 5.0; UD: 1010, Proteins: ++ / 4; Leukocytes: ++ / 4; Hemoglobin: ++ / 4. Sedimentoscopy: RBC (zero), Leukocytes (15 / field) (Table 1). At the physical examination she had BP of 160/110 mmHg and the fundus examination showed flame-shaped hemorrhages, with pathological AV crossing and tortuosity without other alterations. Schistocyte screening was positive, with negative direct Coombs test, and negative serologies for hepatitis B, C and HIV, as well as negative anti-double-stranded DNA, Anti-SSA and Anti-SSB. The renal biopsy was compatible with thrombotic microangiopathy, associated with moderate interstitial fibrosis and acute tubular necrosis (Figs. 4 and 5). The patient persisted with BP peaks and received optimized antihypertensive medication, being discharged without renal function recovery, with persistence of the need for dialysis therapy at discharge. The diagnosis was Thrombotic Microangiopathy (TMA) secondary to cocaine use.

### Case 03

A 25-year-old male patient, without comorbidities, who had been a cocaine user for 5 years had a sudden onset of generalized disabling myalgia (especially in the lower limbs) associated with recent frontotemporal headache, palpitation, dizziness, and a non-measured febrile episode; the patient had used cocaine at the night before symptom onset. The physical examination showed hemorrhagic suffusion, blood pressure of 180/110 mmHg and tachycardia of 110 bpm. The patient was initially treated in another unit, which did not have logistics to dose myoglobin, and also no records regarding the initial value of CPK. Initial laboratory examinations from our hospital admission until 5 days after the admission showed Serology for HIV, hepatitis B and C negative; Creatinine of 13.8 mg/dL; Urea of 259 mg / dL (Table 1), and urgent hemodialysis was performed. CPK was 1731 U/L. During the hospitalization, the patient showed progressive improvement of both hemorrhagic suffusion and elevated BP. He showed improved diuresis and recovery of renal function in 13 days during hospitalization, and dialysis therapy was suspended. The final probable diagnosis was AKI secondary to cocaine-induced rhabdomyolysis**.**

## Discussion and conclusions

The pathophysiological basis of cocaine-related kidney injury involves renal hemodynamic changes; glomerular matrix synthesis and degradation; oxidative stress and renal atherogenesis induction, which may result in several renal manifestations, being associated with, acute glomerulonephritis, interstitial nephritis and rhabdomyolysis [6].

**Fig. 6:**
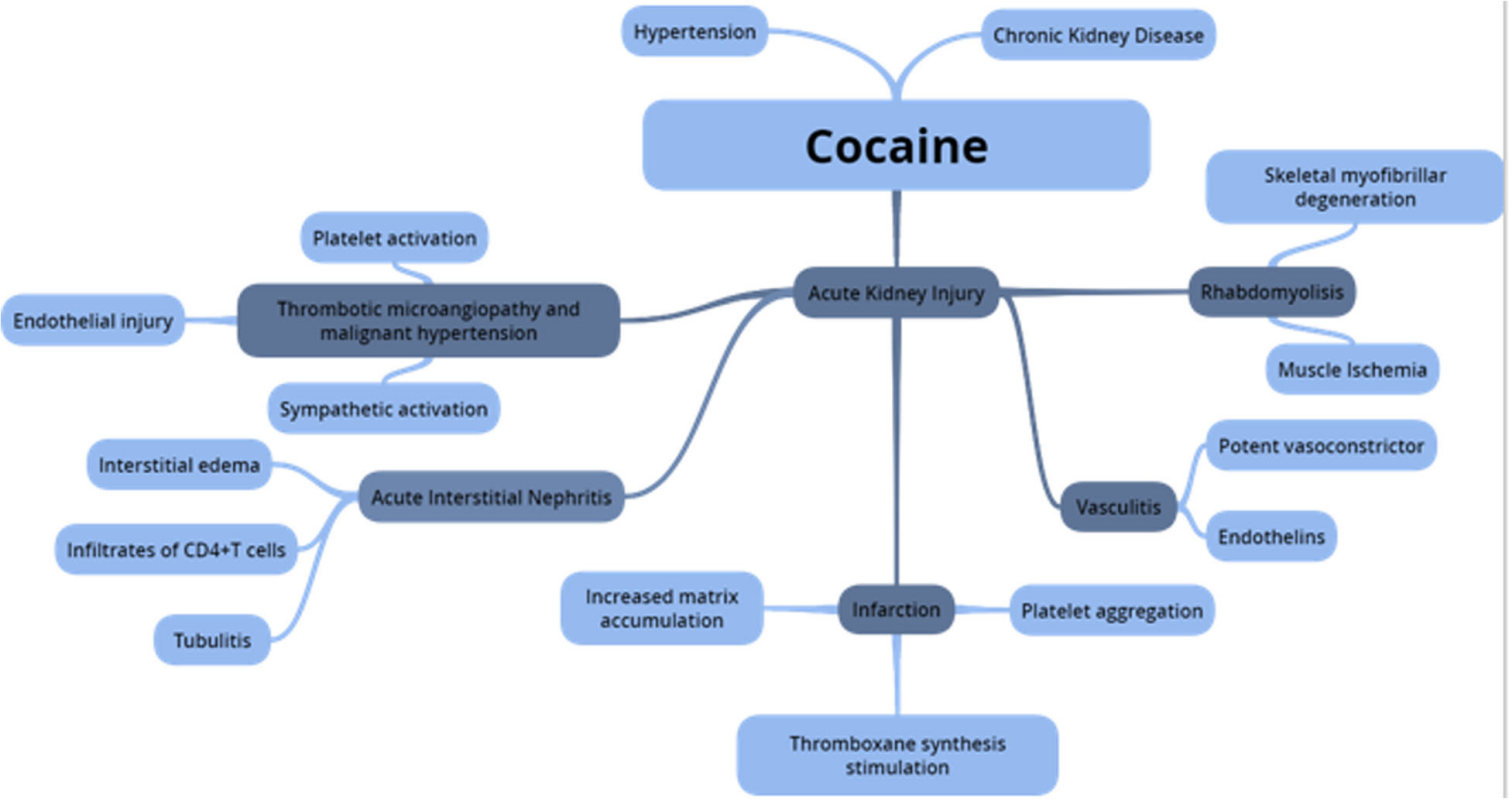
Pathogenesis of renal damage induced by cocaine use

Cocaine stimulates the sympathetic nervous system through distinct mechanisms that include inhibiting catecholamine uptake at the sympathetic nerve terminals, as well as the release of epinephrine and norepinephrine by the adrenal medulla [6]. At the renal level, in addition to this potent vasoconstrictor effect, modifications in the structure and hemodynamics of this organ seem to occur. Fibrosis occurs due to excessive activation of the Renin-Angiotensin-Aldosterone System, as well as accelerated atherogenesis. These pathological alterations can occur in three clinical manifestations, which are acute kidney injury, chronic kidney disease and hypertension with different causes (Fig. 6) [7, 8].

A common fact to all cases was the occurrence of acute kidney injury. This entity is defined as a syndrome with rapid loss of renal function with accumulation of nitrogenous slags [9]. The diagnosis necessarily involves analysis of urea and creatinine levels, with oliguria being a little sensitive and specific symptom [10]. It should be emphasized that this injury shows high morbidity and mortality, if not promptly reversed [11].

It is well-known, as previously explained in the introduction, that the systemic spectrum, particularly the renal ones, course with several clinical manifestations [8]. Therefore, there should be a strong clinical suspicion in patients with a history of cocaine abuse, since many of the alterations caused by this substance are common to several other pathologies, hence being a diagnosis of exclusion [5].

The three cases reported here demonstrated several renal manifestations related to cocaine use. In the first case, we reported on a female patient with a positive pauci-immune ANCA-related RPGN (age group consistent with such pathology), who did not have the characteristic diffuse purpuric rash, which usually affects the extremities and ears, with a purpuric and consistent appearance [12, 13]. In spite of that, renal involvement hardly shows clinical manifestations [5]. The renal biopsy, immunofluorescence, and positive C-ANCA are compatible with the diagnosis of Pauci-Immune Rapidly Progressive Glomerulonephritis [14].

An important fact related to the association between cocaine and positive C-ANCA are the ANCA high titers. This fact supports the presence of cocaine-related vasculitis, instead of primary vasculitis [15]. Glomerulonephritis seems to be a synergistic effect of cocaine adulterated by substances such as levamisole [14]. Other effects also caused by levamisole are leukocytoclastic and thrombotic vasculitis [5, 16].

The pathophysiological mechanism of pauci-immune ANCA-related RPGN remains unknown. One of the proposed models suggests that the activation of neutrophils exposes ANCA antigens, which would promote the remaining activation of neutrophils, as well as adhesion and migration through the endothelium with consequent tissue injury [14]. Thus, T-cell activation is prevalent.

The second case reports on a patient with microangiopathy, thrombocytopenia and acute kidney injury secondary to cocaine use. These clinical and laboratory changes correspond to Thrombotic Microangiopathy (TMA), with the kidney being a frequent site of involvement [17, 18]. This syndrome has several etiologies, being related to both hereditary and acquired diseases. Severe hypertension crisis, drug-induced, infections (including Uremic Hemolytic Syndrome (HUS) and thrombotic thrombocytopenic purpura (TTP) are some examples of etiology [17, 19].

The pathophysiology of TMA is also diverse, depending on the related etiology. In the case of TTP corresponds to a deficiency of ADAMST-13, in the case of HUS or atypical HUS corresponds to a deregulation in the complement system and in the case of drug-mediated may correspond to a set of mechanisms [19, 20]. TMA inducing by drugs is rare, and there are reports of different substances related to its occurrence. The pathophysiology is uncertain, being hypotheses ranging from immune changes to endothelial dysregulation of podocytes [21].

Cocaine inducing TMA or similar pictures has few reports in the literature. Patients users of this substance who present a recent history of microangiopathic hemolytic anemia should be in the differential diagnosis of TMA-induced drug. An important aspect in the approach of these patients is to have the dosage of ADAMTS-13 as it allows to exclude TTP frames. For logistic reasons, we do not have the test for the patient of the report 2 [22, 23].

The early diagnosis of TMA is essential, since early measures are required, often requiring early dialysis or immunosuppression [19].

The third case reports on a patient with sudden onset of myalgia, vomiting and nausea, suggesting rhabdomyolysis, which was later confirmed by laboratory tests. This pathology is the most common nephropathy related to cocaine use [21]. Generally, CPK levels > 5000 IU/L are expected in patients with rhabdomyolysis and renal failure. This patient only had CPK levels measured 5 days after admission; thus, it is possible to infer very high values at the clinical picture onset, considering that there is a daily reduction in serum CPK levels of 40 to 50% [24].

The epidemiology of cocaine-induced rhabdomyolysis includes young black male patients [22]. The typical clinical picture consists of delirium, hyperthermia and excitability, which are present in rhabdomyolysis, regardless of the etiology [24].

**Fig. 7:**
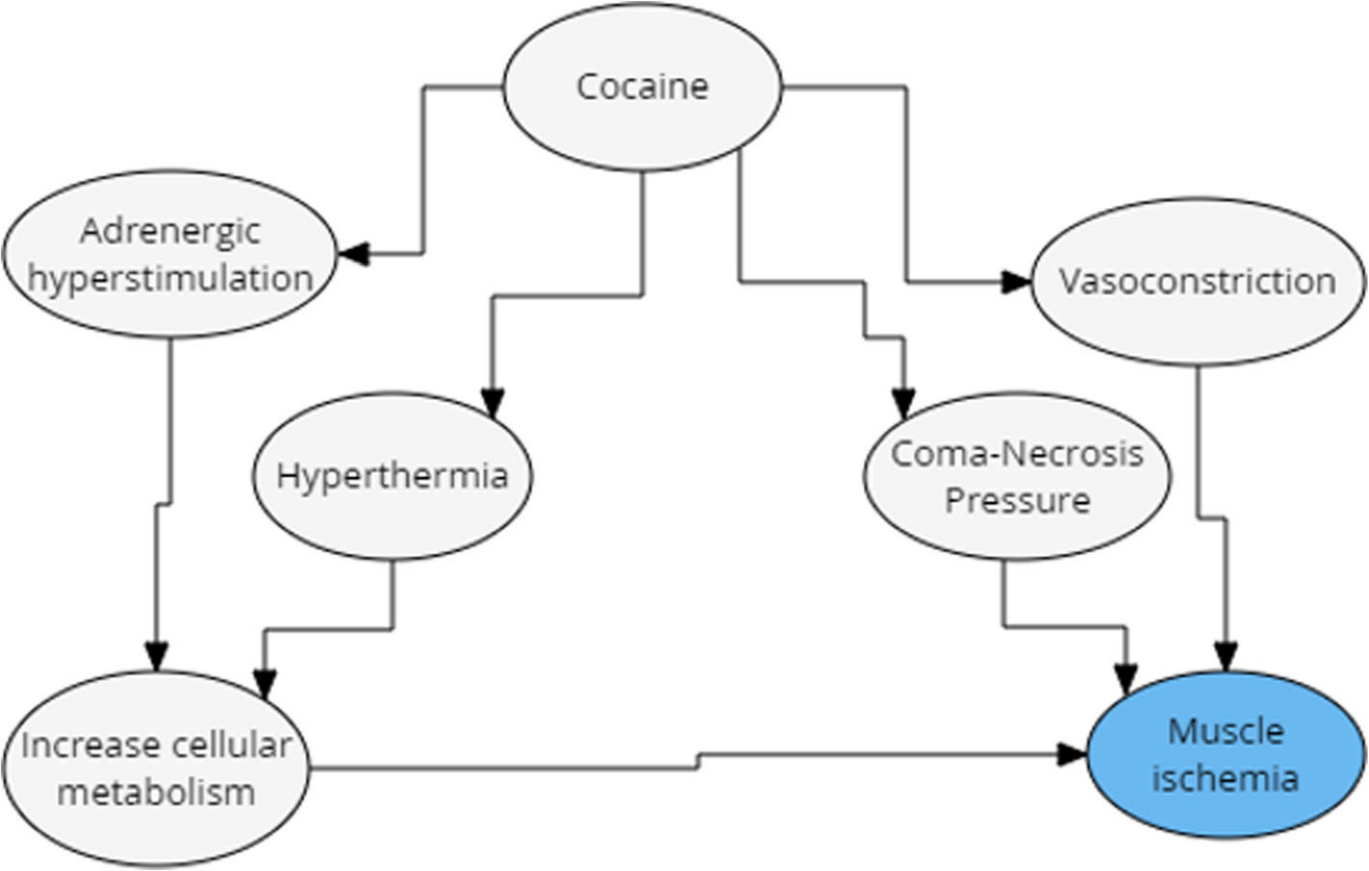
Pathogenesis of rhabdomyolysis induced by cocaine use

The incidence of rhabdomyolysis among cocaine users was 24% in a certain study. Despite the significant prevalence, the pathophysiology of this association remains uncertain. Among the hypotheses used to explain this association are vasoconstriction resulting in muscle ischemia and the direct toxic effects of cocaine (Fig. 7). An important fact is that other etiologies should be ruled out, such as trauma, alcohol consumption and infections, so that it can be inferred that rhabdomyolysis was caused by cocaine use [24].

**Fig. 8:**
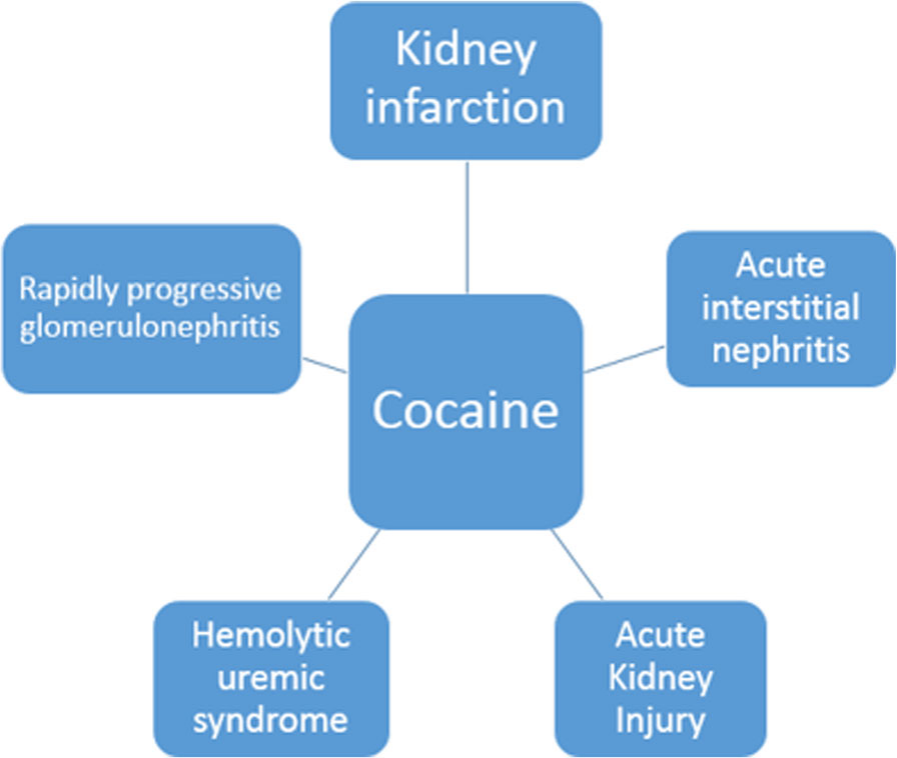
Cocaine use and AKI etiologies

The literature reports other etiologies of cocaine-induced kidney injury (Fig. 8) [6]. Acute interstitial nephritis is one of them, with very few published reports. The epidemiology of these patients consists in male gender individuals aged around 40 years at presentation [23]. The clinical manifestations suggested for these patients is the onset of oliguria with abdominal pain, with dialysis often being necessary. The treatment, despite the etiology, consists in the withdrawal of the inducing agent, with the use of corticosteroids remaining controversial [23].

Finally, renal infarction is another important etiology of cocaine-induced kidney injury. It is also an etiology with few published reports. There seems to be no higher incidence between genders. The exact pathophysiology is unknown; however, as shown in flow chart I, there is platelet aggregation, increased thromboxane production, as well as increased endothelin production, together with an atherogenic effect [8, 25].

The presence of valvular heart disease and atheroembolic events are common risk factors. The clinical manifestations observed in these patients comprise intense and persistent flank pain, associated with nausea and/or vomiting, which may occur regardless of the cocaine administration route [24]. There is no consensus regarding treatment, and basal conditions, such as associated coagulopathies, should be assessed [25].

Therefore, cocaine and its adulterants, especially levamisole, directly and indirectly contribute to renal function and structure alterations, mainly regarding microhemodynamics. Increase in oxidative stress, increased platelet activation, increased production and activation of prostaglandins, increased sympathetic activity and endothelial dysfunction are the main pathophysiological effects caused by cocaine use. Consequently, the spectrum is varied and can be gathered into three groups: hypertension, chronic kidney disease and acute kidney injury, which was the focus of the present study.

In conclusion basically, 05 etiologies of acute kidney injury should always be remembered: rhabdomyolysis, thrombotic microangiopathy, vasculitis, acute interstitial nephritis and renal infarction. Emphasis should be given to rhabdomyolysis due to its higher prevalence. Considering the increasing rates of cocaine use, especially with the use of adulterating substances, these pathologies will likely be increasingly prevalent.

At first, the treatment may not be different, but there are significant differences in the prognosis of these patients, as well as the time of intensive monitoring, among other measures. The scarcity of reported cases suggests underdiagnosis and / or underreporting of cocaine-induced kidney injury and high clinical suspicion is important in patients with risk factors.

The scarcity of reports contributes to the uncertainty regarding several aspects of cocaine-induced nephropathies.

## Abbreviations

aHUS: Atypical hemolytic uremic syndrome
C-ANCA: Cytoplasmic antineutrophil cytoplasmic antibodies
RPGN: Rapidly progressive glomerulonephritis

## References

[1] Haas C, Karila L, Lowenstein W (2009). Cocaine and crack addiction: A growing public health problem. Bull Acad Natl Med.

[2] CHAPTER I Cocaine 35 [Internet]. [cited 2018 Dec 12]. Available from: http://www.unodc.org/doc/wdr2016/WDR_2016_Chapter_1_Cocaine.pdf.

[3] Rigacci R, Madruga CS, Ribeiro M, Pinsky I, Caetano R, Laranjeira R (2014). Addictive behaviors prevalence of cocaine use in Brazil: data from the II Brazilian National Alcohol and Drugs Survey (BNADS). Addict Behav.

[4] Crack Cocaine—Accelerating Use in Brazil | National Institute on Drug Abuse (NIDA) [Internet]. [cited 2018 Dec 13]. Available from: https://www.drugabuse.gov/international/abstracts/crack-cocaine-accelerating-use-in-brazil.

[5] Díaz HÁ, Callejo AIM, Rodríguez JFG, Pazos LR, Buela IG, Barrera AMB (2013). ANCA-positive vasculitis induced by levamisole-adulterated cocaine and nephrotic syndrome: the kidney as an unusual target. Am J Case Rep.

[6] Jaffe JA, Kimmel PL (2006). Chronic nephropathies of cocaine and heroin abuse: a critical review. Clin J Am Soc Nephrol.

[7] Schwartz BG, Rezkalla S, Kloner RA (2010). Cardiovascular effects of cocaine. Circulation.

[8] Goel N, Pullman JM, Coco M (2014). Cocaine and kidney injury: a kaleidoscope of pathology. Clin Kidney J.

[9] Bellomo R, Kellum JA, Ronco C (2012). Acute kidney injury. Lancet.

[10] MacEdo E, Malhotra R, Claure-Del Granado R, Fedullo P, Mehta RL (2011). Defining urine output criterion for acute kidney injury in critically ill patients. Nephrol Dial Transplant.

[11] Kellum JA, Lameire N, Aspelin P, Barsoum RS, Burdmann EA, Goldstein SL (2012). KDIGO clinical practice guideline for acute kidney injury. Kidney Int Suppl.

[12] Roberts JA, Chevez-Barrios P (2015). Levamisole-induced vasculitis: a characteristic cutaneous vasculitis associated with levamisole-adulterated cocaine. Arch Pathol Lab Med.

[13] Morris GW, Mason BC, Harris Sprunger R, Hake Harris H, White LA, Patterson DA (2012). Levamisole-adulterated cocaine: a case series. J Am Board Fam Med.

[14] Rowaiye OO, Kusztal M, Klinger M (2015). The kidneys and ANCA-associated vasculitis: from pathogenesis to diagnosis. Clin Kidney J.

[15] Carlson AQ, Tuot DS, Jen K-Y, Butcher B, Graf J, Sam R (2014). Pauci-immune glomerulonephritis in individuals with disease associated with levamisole-adulterated cocaine. Medicine (Baltimore).

[16] Gazoni FM, Truffa AA, Kawamura C, Guimarães HP, Lopes RD, Sandre LV (2006). Complicações cardiovasculares em usuário de cocaína: relato de caso. Rev Bras Ter Intensiva.

[17] George JN, Charania RS (2013). Evaluation of patients with microangiopathic hemolytic anemia and thrombocytopenia importance of diagnosing microangiopathic hemolytic anemia for decisions on therapy. Semin Thromb Hemost.

[18] Brocklebank V, Wood KM, Kavanagh D (2018). Thrombotic microangiopathy and the kidney. Clin J Am Soc Nephrol.

[19] Barbour T, Johnson S, Cohney S, Hughes P (2012). Thrombotic microangiopathy and associated renal disorders. Nephrol Dial Transplant.

[20] Shatzel JJ, Taylor JA (2017). Syndromes of thrombotic microangiopathy. Med Clin North Am.

[21] Al-Nouri ZL, Reese JA, Terrell DR, Vesely SK, George JN (2015). Drug-induced thrombotic microangiopathy: a systematic review of published reports. Blood.

[22] Huttinger KJ, Kirrmann H (2014). Cocaine-induced microangiopathic hemolytic anemia mimicking idiopathic thrombotic thrombocytopenic purpura: a case report and review of the literature. J Clin Apher.

[23] Volcy J, Nzerue CM, Oderinde A, Hewan-Iowe K (2000). Cocaine-induced acute renal failure, hemolysis, and thrombocytopenia mimicking thrombotic thrombocytopenic purpura. Am J Kidney Dis.

[24] Welch RD, Todd K, Krause GS (1991). Incidence of cocaine-associated rhabdomyolysis. Ann Emerg Med.

[25] Bemanian S, Motallebi M, Nosrati SM (2005). Cocaine-induced renal infarction: report of a case and review of the literature. BMC Nephrol.

